# Global, regional and national burden of orofacial clefts from 1990 to 2019: an analysis of the Global Burden of Disease Study 2019

**DOI:** 10.1080/07853890.2023.2215540

**Published:** 2023-05-26

**Authors:** Dawei Wang, Boyu Zhang, Qi Zhang, Yiping Wu

**Affiliations:** Department of Plastic Surgery, Tongji Hospital of Tongji Medical College of Huazhong University of Science and Technology, Wuhan, China

**Keywords:** Orofacial clefts, global burden, incidence, deaths, DALYs

## Abstract

**Background:**

Orofacial clefts are the most common congenital malformation, but the global burden and trends of orofacial clefts have not been comprehensively analysed. The aim of this study was to assess the global incidence, deaths and disability-adjusted life years (DALYs) of orofacial clefts by countries, regions, sex and sociodemographic index (SDI) from 1990 to 2019.

**Methods:**

The data on orofacial clefts were obtained from the Global Burden of Disease Study 2019. The incidence, deaths and DALYs were analysed by countries, regions, sex and SDI. Age-standardized rates and estimated annual percentage change (EAPC) were calculated to evaluate the burden and temporal trend of orofacial clefts. The association between EAPC and the human development index was assessed.

**Results:**

Globally, the incidence, deaths and DALYs of orofacial clefts decreased from 1990 to 2019. The high SDI region showed the biggest downward trend in incidence rate from 1990 to 2019, along with the lowest age-standardized death rate and DALY rate. Some countries, such as Suriname and Zimbabwe, experienced increased death rate and DALY rate over time. The age-standardized death rate and DALY rate were negatively associated with the level of socioeconomic development.

**Conclusion:**

Global achievement is evident in the control of the burden of orofacial clefts. The future focus of prevention should be on low-income countries, such as South Asia and Africa, by increasing healthcare resources and improving quality.

**KEY MESSAGES**
- This is the most recent estimate of the global epidemiology of orofacial clefts, with some countries not previously assessed.
- The global burden of orofacial clefts showed downward trends from 1990 to 2019; however, some low-income countries are still suffering from increasing burdens.
- Effective measures should be taken to reduce the burden of orofacial clefts in the uncontrolled regions.

## Introduction

Orofacial clefts are the most common congenital craniofacial defects, which pose adverse effects on health, quality of life, personal self-esteem and social behaviour [1,2]. The treatment of orofacial clefts requires high medical costs; the lifetime treating cost of one individual has been estimated to be $100,000 in the United States [3]. The most common orofacial clefts are cleft lip only, cleft palate only, and cleft lip with cleft palate [4]. The majority of orofacial clefts are non-syndromic without any other birth defects or cognitive abnormality, whereas the syndromic type is associated with other birth defects or cognitive abnormalities [4,5].

The aetiology of orofacial clefts is complex, relating to different embryological origins and times of development [6,7]. The failure of the formation of the primary palate leads to a cleft lip, while a cleft palate arises from the failed formation of the secondary palate [4]. The cause of orofacial clefts can be considered as the interaction between genetic alterations and environmental factors [8,9]. Recently, many candidate genes and loci have been demonstrated to be associated with the occurrence of orofacial clefts [10–­12]. The environmental factors, including smoking, alcohol consumption, dietary and vitamin deficiencies, parental age, environmental toxins and socioeconomic status, may also affect the presence of orofacial clefts [13–17].

Orofacial clefts affect approximately 1 in 700 live births worldwide, while the epidemiological data of orofacial clefts vary widely with geographic location, ethnic backgrounds, sex and socioeconomic status [4,5]. Studies have shown that the prevalence of orofacial clefts is higher in Asians compared with Whites and Blacks, with males affected more than females [18,19]. The risk of orofacial cleft has also been reported to increase with parental age [20]. The detailed knowledge of epidemiology at the global level is critical to the prevention of orofacial clefts and the allocation of medical resources. However, the previous studies focused on more developed countries or regions, and large gaps existed in registry data from low-income countries [21,22]. There are few comprehensive studies to assess the global burden of orofacial clefts for all countries and regions [23]. In addition, multi-level analysis is needed to identify the temporal trends and influencing factors on the burden of orofacial clefts, which can provide a reference for the development of prevention strategies.

The Global Burden of Disease (GBD) study provides epidemiological data on 369 diseases (including orofacial clefts) in 204 countries and territories worldwide [24,25]. To investigate the global, regional and national burden of the orofacial clefts, we analysed the incidence, deaths and disability-adjusted life years (DALYs) data from the GBD study 2019 by countries, regions, sex and sociodemographic index (SDI) value. This study provides a comprehensive understanding on the current burden of orofacial clefts to facilitate medical practice and policy making.

## Methods

The epidemiological data on orofacial clefts were collected from the GBD 2019 study (https://vizhub.healthdata.org/gbd-results/). The data included the incidence, deaths and DALYs of orofacial clefts from 1990 to 2019 in 204 countries and territories. The definition of orofacial clefts in the GBD study referred to the ICD 9 and ICD 10 codes. The 204 countries and territories were separated into 21 regions in terms of geography. Moreover, these countries and territories were classified into five regions in terms of SDI, including high, high-middle, middle, low-middle and low. The SDI is a composite indicator reflecting the social and economic development of a location based on the assessment of incomes per capita, education level and fertility rates. SDI values scaled from 0 to 1, with larger values representing the more developed country.

The annual number of incident cases, death and DALYs were obtained from the website. Introduced by the World Bank and the WHO, the DALY is increasingly used for assessing the disease burden on individual health status [26,27]. DALY is a summary measure of the years lived with disability and the years of life lost. DALY is a positive value, with larger values reflecting more severe loss of healthy life caused by the disease. The age-standardized rates (ASR) of incidence, death, and DALYs were calculated to describe the disease burden. The ASR indicates the number of incident cases, death or DALYs per 100,000 population with adjusted for population age differences [28]. ASR values scaled from 0 to 100,000, with larger values indicating higher morbidity, mortality or DALY rates. The associations between the SDI and ASR were calculated using Pearson’s correlation analysis.

The estimated annual percentage change (EAPC) was calculated to reflect the temporal trends of the ASR of incidence, death and DALYs. The natural logarithm of the regression line fitted to the ASR, represented by the formula y = a + bx + c. In this formula, x is the calendar year, and y represents ln (ASR). The EAPC was calculated as 100*(exp[b] − 1), and its 95% confidence interval (CI) was also obtained. If the EAPC and the lower limit of 95% are both positive, the ASR is considered to be on an increasing trend. Conversely, the ASR is considered to be on a decreasing trend if the EAPC and the upper limit of 95% CI are both negative. The Human Development Index (HDI) indicates the quality and availability of healthcare in each country, which is available to the World Bank [29]. The association between each EAPC and HDI was evaluated using Pearson’s correlation analysis.

All statistical analyses and data visualization were conducted in the R software (Version 3.4.4, R core team). The ‘maps’ package was used to generate the world map, and the ‘ggplot2’ package was to create statistical figures. A *p* value less than 0.05 was considered statistically significant.

## Results

### Incidence of orofacial clefts

Globally, the incident number of orofacial clefts decreased from 237,258 cases (95% CI: 155,983–364,211 cases) in 1990 to 192,708 cases (95% CI: 125,226–294,619 cases) in 2019, corresponding to a decrease of 19% (−23% to −14%). Similarly, the global ASR of incidence of orofacial clefts decreased from 3.61 (95% CI: 2.37–5.54) per 100,000 in 1990 to 2.98 (95% CI: 1.93–4.55) per 100,000 in 2019 with an EAPC of −0.69 (95% CI: −0.81 to −0.58).

**Figure 1.**
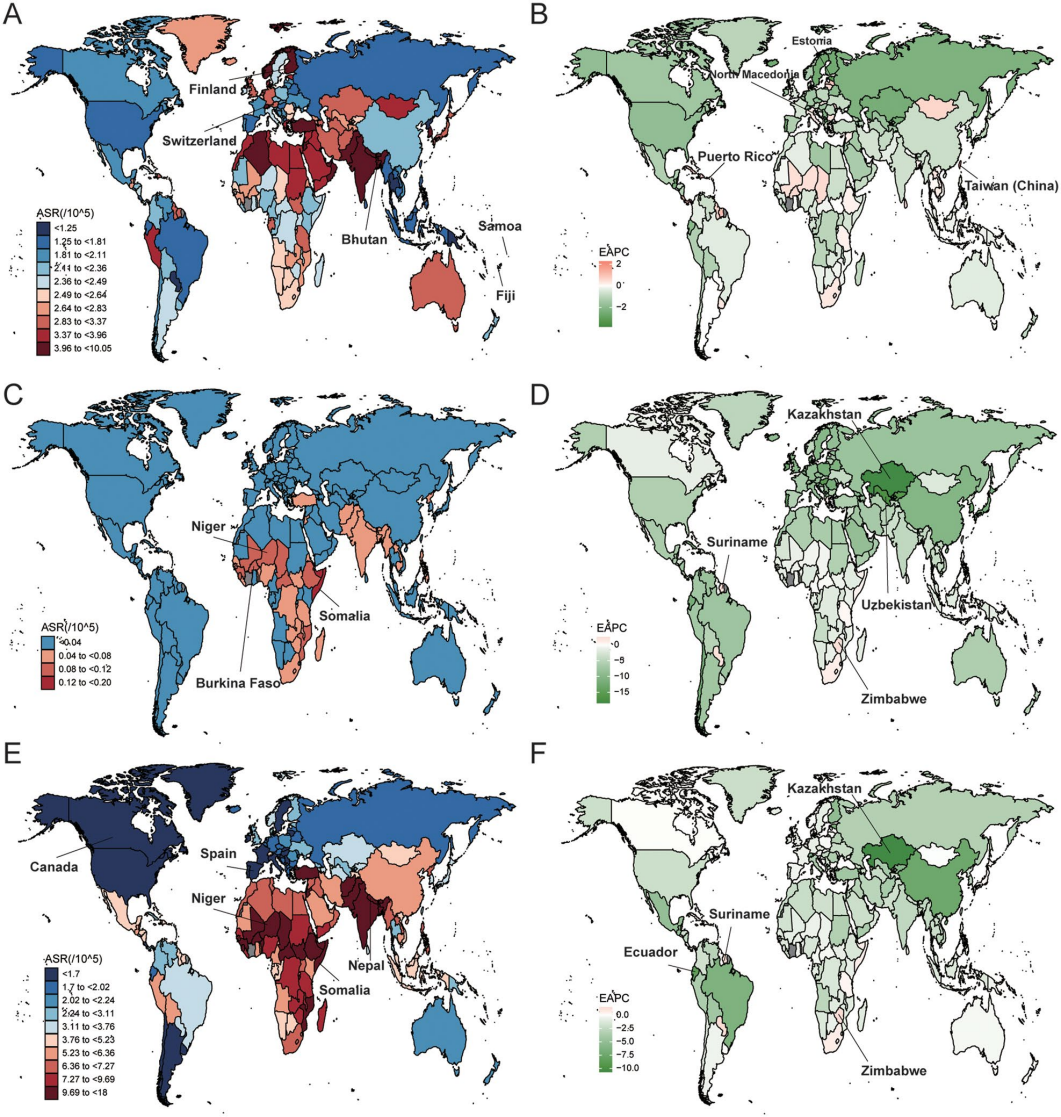
ASR and EAPC of orofacial clefts across 204 countries/territories. (A) ASR of incidence in 2019; (B) EAPC in ASR of incidence from 1990 to 2019; (C) ASR of deaths in 2019; (D) EAPC in ASR of deaths from 1990 to 2019; (E) ASR of DALYs in 2019; (F) EAPC in ASR of DALYs from 1990 to 2019. ASR: age-standardized rate; EAPC: estimated annual percentage changes; DALYs: disability-adjusted life years.

In 2019, the ASR of incidence across the 204 countries was the highest in Switzerland (6.14/100,000, 95% CI: 3.99–9.75/100,000), followed by Bhutan, Finland, while the lowest in Samoa (0.92/100,000, 95% CI: 0.65–1.37/100,000) and Fiji (Figure 1(A)). The EAPCs in the incidence were the highest in the Taiwan (China) (2.28, 95% CI: 1.88–2.68) and Puerto Rico (1.44, 95% CI: 1.22–1.67), while the lowest in North Macedonia (−3.81, 95% CI: −4.26 to −3.36) and Estonia (−3.16, 95% CI: −3.5 to −2.82) (Figure 1(B)).

**Figure 2.**
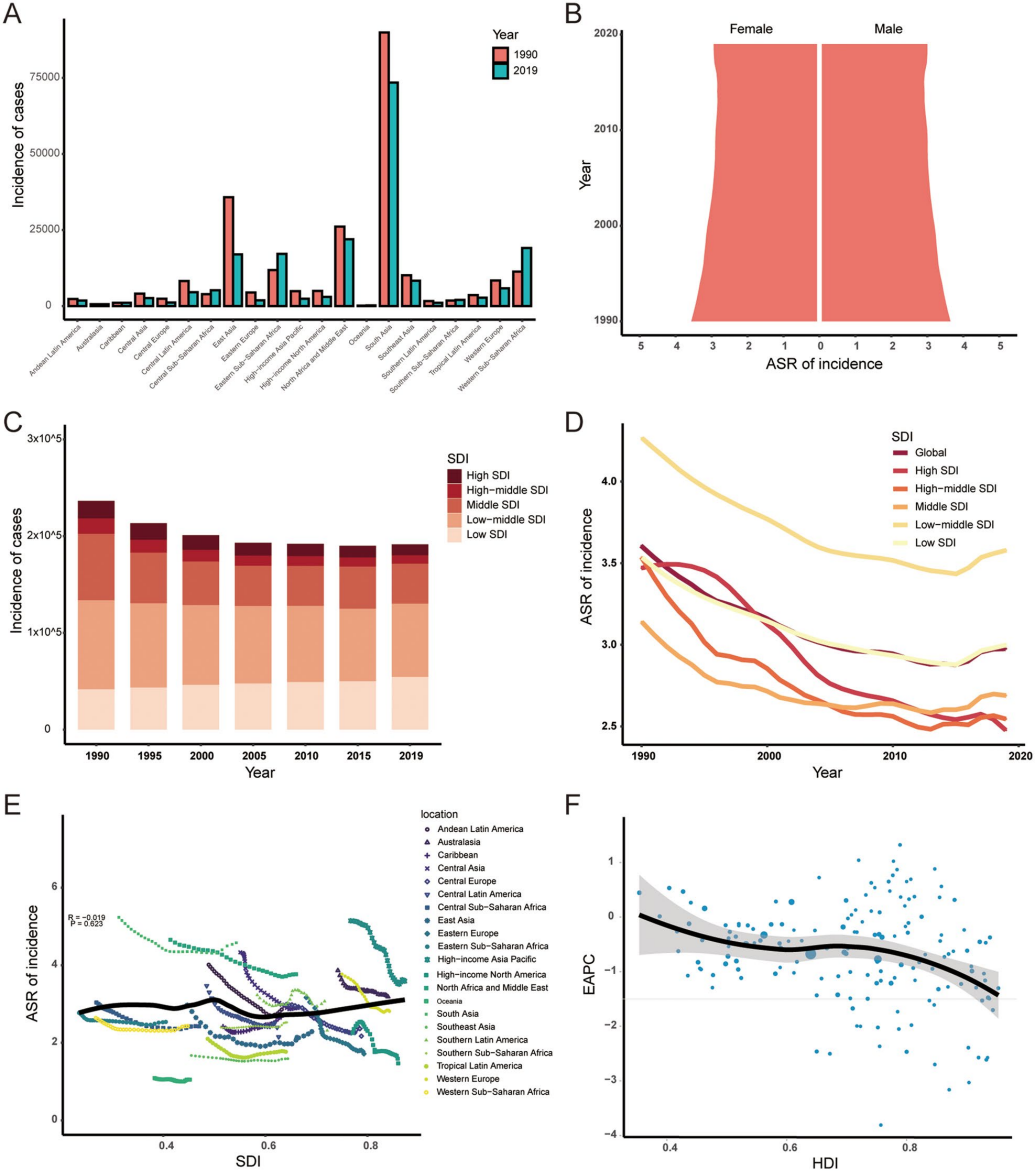
The incidence of orofacial clefts. (A) The number of incident cases in 21 regions from 1990 to 2019; (B) the ASR of incidence in males and females from 1990 to 2019; (C) changes in incident cases in five SDI regions from 1990 to 2019; (D) changes in ASR of incidence in five SDI regions from 1990 to 2019; (E) association between SDI and ASR of incidence in 21 regions; (F) association between HDI and EAPC in ASR of incidence. ASR: age-standardized rate; EAPC: estimated annual percentage changes; SDI: sociodemographic index; HDI: human development index.

In term of geographic regions, the number of incident cases has decreased in most regions, except Oceania (78%, 95% CI: 65–92%), Western Sub-Saharan Africa (69%, 95% CI: 62–75%), Eastern Sub-Saharan Africa (45%, 95% CI: 36–52%), Central Sub-Saharan Africa (33%, 95% CI: 13–52%) and Southern Sub-Saharan Africa (14%, 95% CI: 6–24%) (Supplementary Table S2, Figure 2(A)). In 2019, the ASR of incidence was the highest in South Asia (4.58/100,000, 95% CI: 2.98–7.27/100,000) and the lowest in Oceania (1.05/100,000, 95% CI: 0.74–1.54/100,000). The ASR of incidence also showed decreasing trend in all regions, except Caribbean (EAPC, 0.45, 95% CI: 0.33–0.56) and Southern Sub-Saharan Africa (EAPC, 0.13, 95% CI: 0.09–0.17).

The ASR of incidence from 1990 to 2019 was slightly higher in males than in females (Figure 2(B)). The low-middle SDI region had the largest incidence cases in 1990 (91,953, 95% CI: 56,482–146,041) and 2019 (75,673, 95% CI: 48,716–118,661) (Figure 2(C)). Also, the low-middle SDI region held the highest ASR both in 1990 (4.27, 95% CI: 2.7–6.67) and in 2019 (3.58, 95% CI: 2.31–5.63) (Figure 2(D)). The ASR of incidence decreased in all SDI regions from 1990 to 2019, and the high SDI region showed the biggest downward trend (EAPC: −1.37, 95% CI: −1.49 to −1.25) (Figure 2(D)). There was no significant correlation between ASR of incidence and SDI (ρ = −0.019, *p* = 0.623) (Figure 2(E)). The EAPC in ASR of incidence was found to be negatively correlated with the HDI value (ρ = −0.248, *p* = 0.002) (Figure 2(F)).

### Deaths of orofacial clefts

Globally, the number of deaths due to orofacial clefts decreased from 11,778 cases (95% CI: 6961–16,984 cases) in 1990 to 2769 cases (95% CI: 1659–5437 cases) in 2019, corresponding to a decrease of 76% (−86% to −60%). Similarly, the global ASR of deaths of orofacial clefts decreased from 0.18 (95% CI: 0.11–0.26) per 100,000 in 1990 to 0.04 (95% CI: 0.03–0.08) per 100,000 in 2019 with an EAPC of −5.16 (95% CI: −5.3 to −5.03).

In 2019, the ASR of deaths across the 204 countries was the highest in Somalia (0.17/100,000, 95% CI: 0.02–0.77/100,000), followed by Burkina Faso (0.12/100,000, 95% CI: 0.02–0.45/100,000) and Niger (0.12/100,000, 95% CI: 0.01–0.51/100,000) (Figure 1(C)). The ASR of deaths decreased fastest in Kazakhstan (EAPC: −18.45, 95% CI: −20.33 to −16.53) and Uzbekistan (EAPC: −17.28, 95% CI: −19.73 to −14.75) (Figure 1(D)). The EAPCs in the deaths were the highest in Suriname (2.52, 95% CI: 1.13–3.93) and Zimbabwe (2.12, 95% CI: 1.65–2.59).

**Figure 3.**
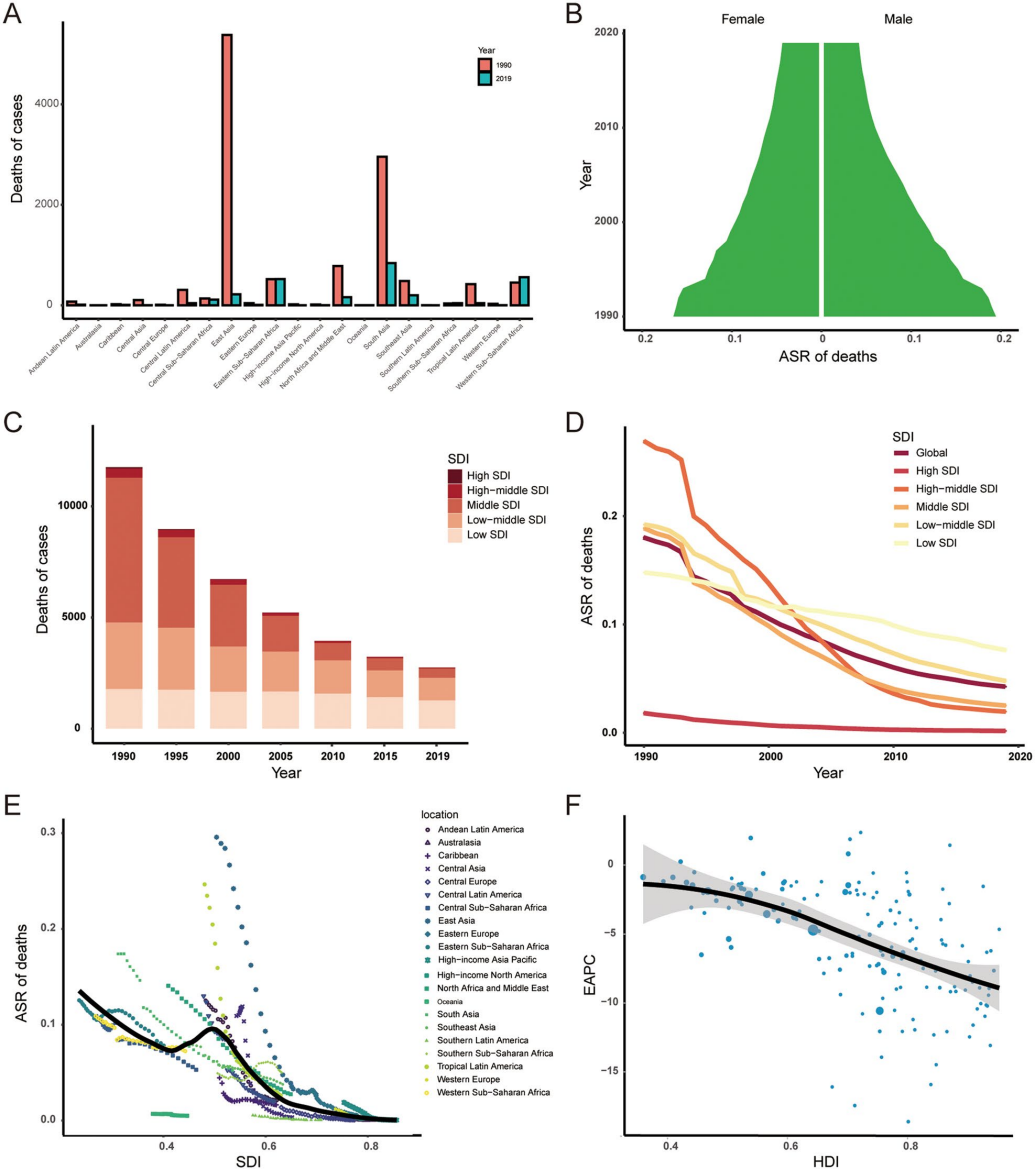
The deaths of orofacial clefts. (A) The number of deaths in 21 regions from 1990 to 2019; (B) The ASR of deaths in males and females from 1990 to 2019; (C) Changes in deaths in five SDI regions from 1990 to 2019; (D) Changes in ASR of deaths in five SDI regions from 1990 to 2019; (E) Association between SDI and ASR of deaths in 21 regions; (F) Association between HDI and EAPC in ASR of deaths. ASR: age-standardized rate; EAPC: estimated annual percentage changes; SDI: sociodemographic index; HDI: human development index.

In terms of geographic regions, the number of deaths has decreased in most regions, except Western Sub-Saharan Africa (24%, 95% CI: −32% to 164%), Southern Sub-Saharan Africa (11%, 95% CI: −40% to 94%) and Eastern Sub-Saharan Africa (1%, 95% CI: −51% to −128%) (Supplementary Table S2, Figure 3(A)). In 2019, the ASR of deaths was the highest in Eastern Sub-Saharan Africa (0.08/100,000, 95% CI: 0.02–0.21/100,000) and Western Sub-Saharan Africa (0.07/100,000, 95% CI: 0.02–0.19/100,000), while no death of orofacial clefts was found in the High-income Asia Pacific, Central Europe, Western Europe, Southern Latin America, High-income North America, Australasia, Central Asia and Oceania. The ASR of deaths showed decreasing trend in all regions, except Southern Sub-Saharan Africa (EAPC: 1.04, 95% CI: 0.62–1.47).

The ASR of deaths from 1990 to 2019 was slightly higher in males than in females (Figure 3(B)). The middle SDI region had the most deaths cases in 1990 (3895, 95% CI: 2482–6011), while the low SDI region had the most deaths cases in 2019 (1359, 95% CI: 519–3393) (Figure 3(C)). The high SDI region held the lowest ASR both in 1990 (0.02, 95% CI: 0.01–0.03) and in 2019 (0, 95% CI: 0–0) (Figure 3(D)). The ASR of deaths decreased in all SDI regions from 1990 to 2019, and the high-middle SDI region showed the biggest downward trend (EAPC: −9.83, 95% CI: −10.26 to −9.4) (Figure 3(D)). There was a negative correlation between ASR of deaths and SDI (ρ = −0.606, *p* < 0.001) (Figure 3(E)). The EAPC in ASR of deaths was found to be negatively correlated with the HDI value (ρ = −0.547, *p* < 0.001) (Figure 3(F)).

### DALYs of orofacial clefts

Globally, the DALYs of orofacial clefts decreased from 1,246,072 years (95% CI: 806,662–1,749,233 years) in 1990 to 529,759 years (95% CI: 362,493–798,420 years) in 2019, corresponding to a decrease of 57% (−72% to −38%). Similarly, the global ASR of DALYs of orofacial clefts decreased from 19.63 (95% CI: 12.85–27.44) per 100,000 in 1990 to 7.51 (95% CI: 5.1–11.57) per 100,000 in 2019 with an EAPC of −3.44 (95% CI: −3.6 to −3.27).

In 2019, the ASR of DALYs across the 204 countries was the highest in Somalia (18/100,000, 95% CI: 4.51–71.6/100,000), followed by Niger, Nepal, while the lowest in Canada (0.89/100,000, 95% CI: 0.56 to 1.32/100,000) and Spain (Figure 1(E)). The EAPCs in the DALYs were the highest in the Suriname (1.49, 95% CI: 0.98–2.01) and Zimbabwe (1.43, 95% CI: 1.11–1.76), while the lowest in Kazakhstan (−10.7, 95% CI: −12.07 to −9.3) and Ecuador (–8.6, 95% CI: −9.43 to −7.76) (Figure 1(F)).

**Figure 4.**
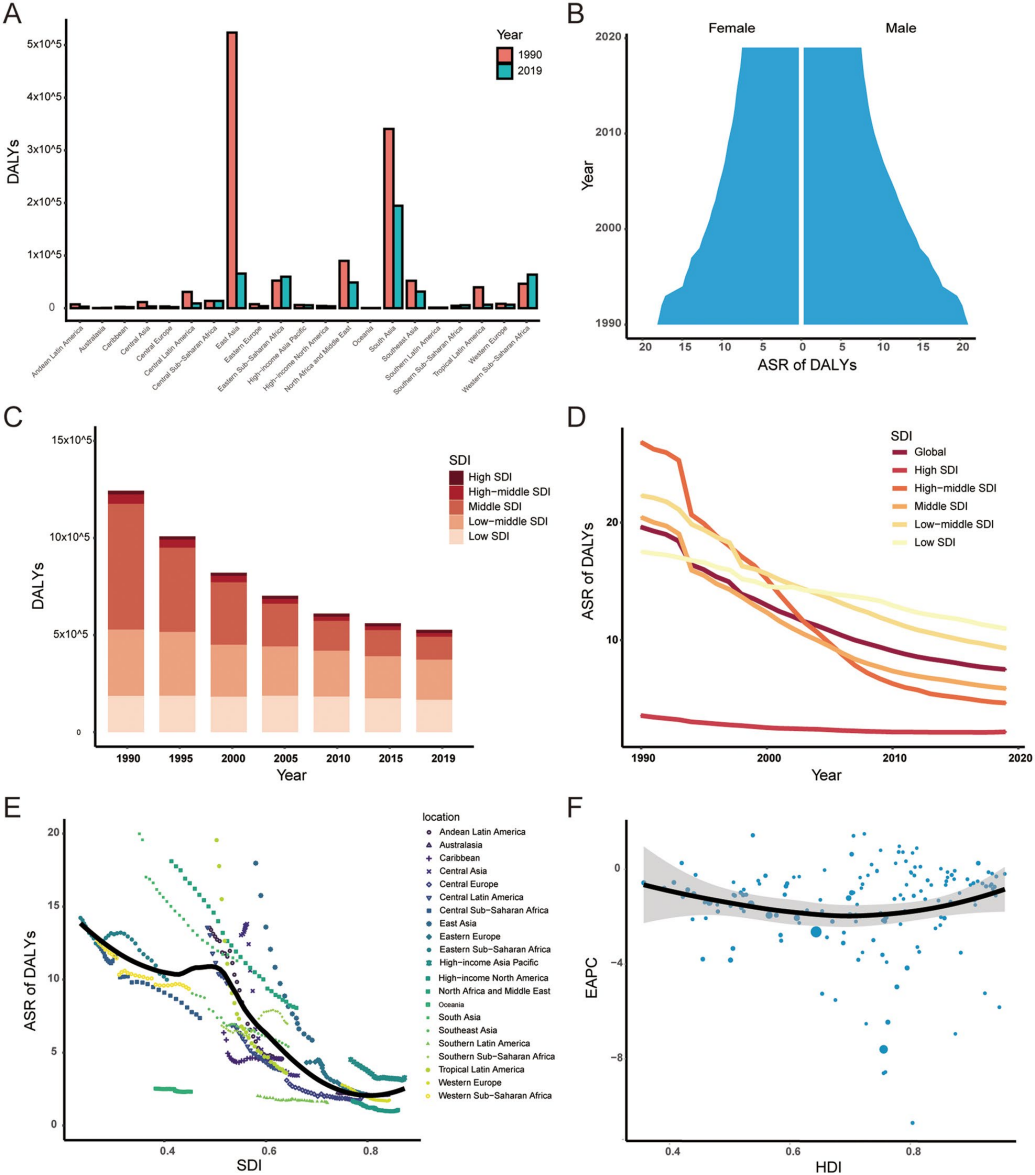
The DALYs of orofacial clefts. (A) The number of DALYs in 21 regions from 1990 to 2019; (B) The ASR of DALYs in males and females from 1990 to 2019; (C) Changes in DALYs in five SDI regions from 1990 to 2019; (D) Changes in ASR of DALYs in five SDI regions from 1990 to 2019; (E) Association between SDI and ASR of DALYs in 21 regions; (F) Association between HDI and EAPC in ASR of DALYs. DALYs: disability-adjusted life years; ASR: age-standardized rate; EAPC: estimated annual percentage changes; SDI: sociodemographic index; HDI: human development index.

In terms of geographic regions, the number of DALYs has decreased in most regions, except Oceania (77%, 95% CI: 20–139%), Western Sub-Saharan Africa (38%, 95% CI: −21% to 148%), Australasia (34%, 95% CI: 12–58%), Southern Sub-Saharan Africa (22%, 95% CI: −21% to 78%) and Eastern Sub-Saharan Africa (14%, 95% CI: −31% to 119%) (Supplementary Table S2, Figure 4(A)). In 2019, the ASR of DALYs was the highest in South Asia (11.29/100,000, 95% CI: 7.08–17.2/100,000) and the lowest in High-income North America (1.07/100,000, 95% CI: 0.7–1.53/100,000). The ASR of DALYs also showed decreasing trend in all regions, except Southern Sub-Saharan Africa (EAPC: 0.69, 95% CI: 0.42–0.96).

The ASR of DALYs from 1990 to 2019 was slightly higher in males than in females (Figure 4(B)). The middle SDI region had the largest DALYs in 1990 (410,635, 95% CI: 277,661–603,137), while low SDI region had the largest DALYs in 2019 (169,948, 95% CI: 88,881–347,052) (Figure 4(C)). The high SDI region held the lowest ASR both in 1990 (3.59, 95% CI: 2.61–4.73) and in 2019 (2.2, 95% CI: 1.4–3.16) (Figure 4(D)). The ASR of DALYs decreased in all SDI regions from 1990 to 2019, and the high-middle SDI region showed the biggest downward trend (EAPC: −6.63, 95% CI: −6.95 to −6.31) (Figure 4(D)). The ASR of DALYs was found to be negatively correlated with the SDI (ρ= −0.630, *p* < 0.001) (Figure 1(E)). There was no significant correlation between EAPC and HDI value (ρ = −0.007, *p* = 0.924) (Figure 4(F)).

## Discussion

Orofacial clefts present as congenital malformations of the lip, palate or both, causing a heavy burden on the individual, family and society [30]. In this study, we analysed the incidence, deaths, and DALYs of orofacial clefts and their temporal trends from 1990 to 2019. Globally, the ASRs of incidence, deaths and DALYs decreased from 1990 to 2019, demonstrating the effectiveness of current preventive measures. The disease burden is uneven worldwide, however, some countries are still suffering from the increasing burdens, such as Suriname and Zimbabwe.

In this study, the burden of orofacial clefts varied by SDI levels, as the SDI index reflects per capita income, total fertility rates and average years of education. We found that the high SDI region showed the least ASRs of deaths and DALYs, as well as the biggest downward trend in incidence. In 2019, the low-middle region suffered the highest ASR of incidence, and the low region suffered the highest ASR of deaths and DALYs. Moreover, the SDI value was negatively correlated with the ASRs of deaths and DALYs in 21 geographical regions. The difference between high SDI region and low SDI region may be due to factors such as ethnic background, nutritional intake, environmental factors, prenatal screening and healthcare quality.

Previous studies have shown that the prevalence of orofacial clefts varies across ethnic groups, with approximately 1 in 500 individuals of Asian populations, 1 in 1000 in European populations and 1 in 2500 in African populations [18]. In our study, South Asia had both the most incident cases and the highest ASRs of incidence and DALYs. South Asia is the most populous and densely populated region in the world, and also one of the poorest in the world [31]. Besides, the Sub-Saharan Africa region had the highest ASR of deaths as well as the second highest ASR of DALYs, mainly due to poverty and poor health care [32]. Previous reports of birth prevalence of orofacial clefts vary considerably from different African populations [33,34]. Similarly, we found the ASR of incidence was from 2.34 per 100,000 in Ethiopia to 4.13 per 100,000 in Algeria in 2019. Somalia had the highest ASRs of deaths and DALYs in 2019, though the ASR of incidence is not high among African countries, reflecting the weakness of treatment for orofacial clefts in Somalia. A similar situation occurred in other African countries, such as Niger, and Burkina Faso.

Deficient nutrition intake, such as folic acid and zinc, is associated with the occurrence of orofacial clefts [4]. Studies have shown a significantly lower risk of orofacial clefts in the offspring of women taking folic acid-containing supplements before and during pregnancy [13]. Similarly, a study demonstrated the dose-dependent relationship between zinc concentrations in plasma and a lower risk of orofacial clefts [35]. In low SDI regions, pregnant women cannot receive an adequate supply of nutrients, which may be related to the high incidence in these regions. The contributing factors, such as smoking, alcohol consumption, drugs and air pollution, can increase the risk of orofacial clefts in newborns [36,37]. Previous GBD studies have shown a high prevalence of tobacco use in South Asia, which corresponds to the high incidence of orofacial clefts in this region [38]. Alcohol consumption is a major risk factor for the global burden of disease, with a higher proportion of drinkers in European countries [39]. Despite high levels of income and medical care, some European countries, such as Finland and Norway, had a high incidence of orofacial clefts.

Prenatal screening plays a critical role in reducing the global burden of congenital diseases [40]. As high SDI regions tend to have a higher rate of prenatal screening than low SDI regions, a more reduced burden of orofacial clefts can be seen in high SDI regions. Moreover, prenatal screening and selective termination of pregnancy can be influenced by health policies, ethnicity, religious beliefs and population awareness. Orofacial clefts can be diagnosed by prenatal ultrasound, and 3D or 4D ultrasound can enhance the accurate diagnosis of craniofacial anomalies [41]. As selective termination of pregnancy is sensitive and controversial, parents can still benefit from prenatal screening to prepare in advance for the birth of a child with orofacial clefts.

A study revealed that the total medical costs for infants with orofacial clefts were about $11 million higher than those for unaffected infants in United States [42]. The treatment of orofacial clefts is a long-term and sequential therapy, requiring the cooperation of dentists, surgeons, plastic surgeons, pediatricians and psychologists [43]. High SDI regions generally have more medical resources to provide high-quality and effective treatment for patients with orofacial clefts, resulting in lower ASRs of deaths, and DALYs. The HDI value indicates the quality and availability of healthcare in nations, which was also negatively correlated with the EAPCs in ASRs of incidence and deaths from 1990 to 2019. Likewise, the size of the surgical workforce has been reported to have a strong negative association with the disease burden of orofacial clefts [23]. Despite the high ASR of orofacial cleft incidence in some developed countries, the ASRs of deaths and DALYs in these countries were still relatively low, such as Finland, Switzerland, Australia and Germany. Conversely, some African countries, such as Niger, Ethiopia and Somalia, suffered a high ASR of DALYs but a relatively low incidence. Therefore, the treatment level of orofacial clefts has a great impact on the disease burden.

As the considerable cost of medical resources required for orofacial clefts, it is necessary to increase healthcare worker training to relieve the disease burden by reducing patient dysfunction and improving quality of life. Additionally, medical information seeking for orofacial clefts is limited among the population, so there is a need to leverage social media to provide population education and support groups for families with orofacial clefts [44]. Similarly, more deprived areas would result in suboptimal follow-up, considering the financial implication of traveling to multiple clinics [45]. Medical resource reallocation should be a feasible way to address the inequality of healthcare provision. Moreover, legislation is essential to reduce the risk factors of orofacial clefts, such as smoking, air pollution and nutrition intake. Studies revealed that smoke-free legislation has reduced the incidence of orofacial clefts in England, Wales and Northern Ireland [46]. Therefore, these measures may be effective in relieving the disease burden of orofacial clefts, including healthcare worker training, population education, healthcare resource allocation and legislation.

Some limitations should be noted in this study. Firstly, the GBD study is largely determined by the quantity and quality of data, however, the epidemiological data on orofacial clefts are lacking in some countries. Secondly, the registration of orofacial clefts may be missed in the low economic regions, leading to an underestimation of the disease burden. Thirdly, the risk factors are not sufficiently identified to explain the regional differences and temporal patterns in the disease burden of orofacial clefts. More data on risk factors are needed for further research. Finally, detailed data on the types of orofacial clefts need to be collected to clarify the distribution of the various types of orofacial clefts.

## Conclusions

The global burden of orofacial clefts showed downward trends from 1990 to 2019, with decreases in the ASRs of incidence, deaths and DALYs worldwide. However, some countries, especially in low-income regions, are still suffering from increasing burdens. The burden of orofacial clefts in South Asia and Africa remains high, compared to other regions of the world. Effective measures should be warranted to control the burden of orofacial clefts in these regions, such as healthcare worker training, population education, medical resource allocation and legislation.

## Ethical approval and patient consent

The institutional review board of Tongji Hospital, Hubei, China, considered that the study did not require approval since it used publicly available data.

## Supplementary Material

Supplemental Material

Supplemental Material

## Data Availability

The data were obtained through an online query tool from the website of GBD 2019 (https://vizhub.healthdata.org/gbd-results/), and no permissions were required to access the data.
